# Additive Functions in Boolean Models of Gene Regulatory Network Modules

**DOI:** 10.1371/journal.pone.0025110

**Published:** 2011-11-21

**Authors:** Christian Darabos, Ferdinando Di Cunto, Marco Tomassini, Jason H. Moore, Paolo Provero, Mario Giacobini

**Affiliations:** 1 Computational Genetics Laboratory, Dartmouth Medical School, Lebanon, New Hampshire, United States of America; 2 Computational Biology Unit, Molecular Biotechnology Center, Department of Genetics Biology and Biochemistry, University of Torino, Torino, Italy; 3 Information Systems Department, Faculty of Business and Economics, University of Lausanne, Lausanne, Switzerland; 4 Department of Animal Production Epidemiology and Ecology, Faculty of Veterinary Medicine, University of Torino, Torino, Italy; University of Maribor, Slovenia

## Abstract

Gene-on-gene regulations are key components of every living organism. Dynamical abstract models of genetic regulatory networks help explain the genome's evolvability and robustness. These properties can be attributed to the structural topology of the graph formed by genes, as vertices, and regulatory interactions, as edges. Moreover, the actual gene interaction of each gene is believed to play a key role in the stability of the structure. With advances in biology, some effort was deployed to develop update functions in Boolean models that include recent knowledge. We combine real-life gene interaction networks with novel update functions in a Boolean model. We use two sub-networks of biological organisms, the yeast cell-cycle and the mouse embryonic stem cell, as topological support for our system. On these structures, we substitute the original random update functions by a novel threshold-based dynamic function in which the promoting and repressing effect of each interaction is considered. We use a third real-life regulatory network, along with its inferred Boolean update functions to validate the proposed update function. Results of this validation hint to increased biological plausibility of the threshold-based function. To investigate the dynamical behavior of this new model, we visualized the phase transition between order and chaos into the critical regime using Derrida plots. We complement the qualitative nature of Derrida plots with an alternative measure, the criticality distance, that also allows to discriminate between regimes in a quantitative way. Simulation on both real-life genetic regulatory networks show that there exists a set of parameters that allows the systems to operate in the critical region. This new model includes experimentally derived biological information and recent discoveries, which makes it potentially useful to guide experimental research. The update function confers additional realism to the model, while reducing the complexity and solution space, thus making it easier to investigate.

## Introduction

Genes are the central pillar of biological evolution, and therefore of life as we know it. The various genome projects provided us with lists of protein-coding genes which are thought to be fairly complete for many organisms, including human beings. Much less is known about the complex regulatory interactions among genes, responsible for the dynamical processes that allow the genome to shape the organism and its interaction with the environment. These interactions can be represented as genetic regulatory networks (GRNs) representing the regulatory effects of a gene on the others. However interactions within these networks are very subtle, intricate, and ill understood. While GRN sections of a few tens to a few hundreds of genes are known in detail for several organisms, the quality of the data drops dramatically as the network size grows.

Nevertheless, GRNs are currently considered among the most important frontiers of biological sciences and are at the center of tremendous research efforts from the biological community. The increase of quantity and quality of the data generated in the field, fostered by modern high-throughput technologies, is bound to follow the same exponential trend as the gene sequencing did in its time. In the meantime, however, it is possible, and useful, to abstract many details of the individual GRNs in the cell and focus on the system-level properties of the whole network dynamics. This Complex Systems Biology approach, although not immediately applicable to any given particular case, still provides interesting general insight.

An early dynamical model for GRNs was proposed in the late 60's by Kauffman [1] and is known as random Boolean networks (RBNs). This abstraction is very attractive due to its simplicity, yet unveils interesting dynamical phenomena about how the network structure and the gene-gene interactions are at the center of the resilience to transcriptional errors, and yet evolvability of GRNs. The dynamics of RBNs can be discriminated in two main regimes: the *ordered* regime, which is characterized by less changes in the gene activations higher stability to transient faults and lower sensitivity to initial conditions, and the *chaotic* regime, where gene activation changes frequently, resulting in reduced stability and increased evolvability. It has been suggested that cells operate at the border between order and chaos, a regime called *critical* or the *edge of chaos* [2]–[4]. Systems in this regime are capable of exceptional behavior: they show robustness to small perturbations, and yet remain flexible enough to integrate external signals, allowing the system to adapt to new conditions. This is true for both organic [5], [6] and non-organic systems [7] and it is a signature feature of Complex Systems in general. A way to visualize this phase transition into the critical regime makes use of *Derrida plots* [8], which provide a method of classifying RBN systems according to their dynamical behavior.

In previous works [9], [10], we proposed an abstract model of GRNs, based on Kauffman's original RBNs that incorporates modern general knowledge on genes' interactions. In particular we challenged the assumptions of the random network topology and the synchronicity of events. We proposed an update scheme based on gene activation and we used scale-free topologies as proposed by Aldana [11]. At the time, our model's purpose, just as Kauffman's, was not to simulate the GRN of a particular organism, but rather to investigate the general dynamical properties of the ensemble of Boolean networks under specific conditions.

In the present work, we analyze GRN three well characterized biological subnetworks. In a related work, Balleza *et al.* [12] use microarray data and canalizing functions to infer the nature of the gene regulatory network interactions in several organisms. In this work, we take advantage of extra information contained in real-life GRNs, that is, the actual activating or repressing regulating effect of the genes on one another, to propose an extension to the RBN update function proposed by Li et al. [13]. This more biologically sound update function, along with real-life network topologies, fills another gap of the original Kauffman RBN model where the nodes' update functions are completely random.

Building on some very preliminary results [14], we deepen and complete the investigation of the model, propose a numerical way of discriminating the system's regimes that complements Derrida plots, and the slope of the curves at the origin. We also conduct a full study of the systems' attractor space, and we validate the model using a third real-life instance of regulatory network proposed by Li *et al.* [15].

This work is structured as follows: first, we describe the two real-life regulatory networks tackled in our model in the next section. Then we give an overview to RBN models, with particular attention to the identification of their dynamical regime. We also propose a new measure, the criticality distance, that allows to numerically discriminate between systems' regimes by analytically capturing the entire information contained in Derrida plots. The last part of this section is devoted to the description of the different measures that have been proposed in the literature to characterize the state space of RBNs. We also present the new Activator Driven Additive node function, an extension of the RBN update function proposed by Li et al. [13]. The regime characterization of this update function applied to the two real-life regulatory network substrates is discussed. Subsequently, we focus on validating the new update function using a regulatory network where the actual Boolean update functions are known. We describe and study the state space of new RBN models, their dynamical behavior, and their resilience to small perturbations. Finally, the last section discusses the results obtained and outlines possible future lines of research.

## Methods

### Yeast and Embryonic Stem Cells Regulatory Networks

**Figure 1:**
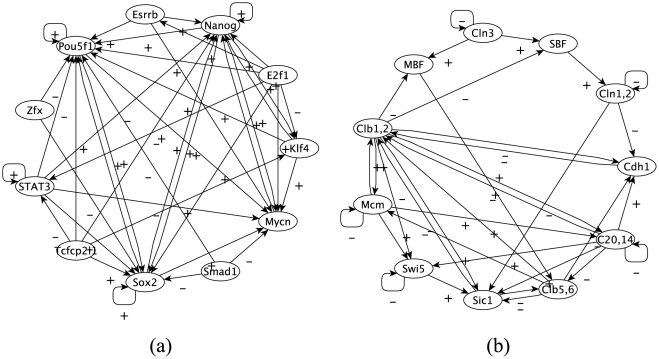
Genetic Regulatory Networks. A representation of (a) the transcriptional regulatory network in ES cells and (b) the yeast cell-cycle regulatory network. Arrows point from transcription factor to the target gene. Signs 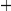 (respectively 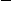) represent activating (respectively repressing) links.

In this section, we give details on the two cases of real-life regulatory networks used in our model. The first one, proposed by Chen *et al.* [16], is a part of the transcriptional regulatory network of embryonic stem (ES) cells inferred from ChIP-seq binding assays and from gene expression changes during differentiation. The activating vs repressing character of the regulatory interactions is not specified in Chen *et al.* [16]. We therefore resorted to a published set of microarray data [17] , available form the GEO database under accession GSE3231, where gene expression profiling of ES cells was performed in a differentiation time-course experiment. We computed the Pearson correlation coefficient of the expression levels of the gene pairs linked in our network, and considered as activating (repressing) the interactions associated to a positive (negative) correlation coefficient. The second one, described by Li *et al.* [13] and as used by Stoll *et al.* [18], is the regulatory network underlying cell cycle in yeast. Both networks have eleven genes. Figure 1 shows these networks, while Table 1 shows some of their statistical properties.

**Table 1:** GRN properties.

	ES cell	Yeast
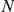	11	11
mean degree	3.72	3.09
enhencer proportion	0.71	0.44

Statistical properties of real-life gene regulatory networks used in this study.

As the networks have too few nodes for a reliable statistical study of their degree distributions, we cannot infer any similitudes of neither the input nor the output degree distributions with either original RBN's random topologies, where the connectivity was a constant, with Erdös-Renyi random networks, nor with Aldana's scale-free input, Poisson output distributions. To reliably establish these degree distributions, one would need to sample at least several tens of nodes for random graphs and many more over several orders of magnitude for scale-free ones, due the long tail of the distribution. However, these data are not currently available. Thus the need to study the dynamical behavior, through Derrida plots or other means, to determine the regime of our models. In this work, we abstract details of the genes themselves, as their individual properties do not have any consequences on the systems dynamics, beyond their activating or repressing effect.

### Random Boolean Networks Modeling

Random Boolean networks were introduced by Kauffman [1], [4] and over the years, numerous other different models have been introduced [19]–[21], but RBNs remain very attractive in their simplicity and ability to include novel concepts. In RBNs, each node represents a gene whose state is a Boolean variable 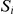 and each directed edge, the influence of a gene on another.

The interconnection topology is considered to be a regular random graph with exactly 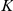 incoming and 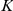 outgoing edges for each gene. A distinct function is given to each node in order to decide state changes according to the state of all in-neighboring genes (i.e. those nodes having an edge directed to the considered target gene). The lookup table describing the update function is randomly generated according to a parameter 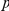 capturing the probability that a gene's state at the next time-step is *active*. The state 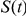 of the system at time 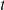 is defined as the ensemble of all the nodes states 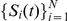. The state changes are fully deterministic, synchronous and instantaneous.

Therefore, these systems, when starting from an arbitrary state 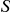 at time 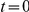, will go through a set of transient states before eventually cycling in a subset of one or more states called an *attractor*. According to Kauffman [1], [4], only attractors that are short and stable to perturbations are of biological interest.

Driving RBN towards a model with biological application potential, a few original assumptions become questionable. Namely, the totally random interaction amongst genes with a fixed connectivity 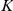 [1] or following a predefined degree distribution, such as scale-free or Poisson [10], [11]. In this work, we will take advantage of the real-life topologies defined in the previous section and use them as the substrate for our Boolean networks. Each gene of the GRN will be replaced by a Boolean variable which specifies whether or not the gene is expressed. In addition, we attach a biologically inspired additive function to each node. In order to investigate the soundness of using a particular update function when modeling a the GRN of a specific organism, we compare the dynamics with those of systems with random update functions.

#### Regimes of RBNs and how to identify them

Original RBNs go through a phase transition at certain values of the fixed degree 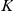 of the nodes and the probability 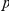 of expressing a gene in the random update function. The critical regime can be achieved by satisfying the equation: 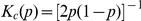. Thus, when the parameter 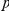 is set to 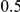, the critical connectivity is achieved as 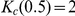. If 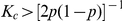, the system will tend to be chaotic, and ordered otherwise. Considering current knowledge about GRNs, some of Kauffman's model properties are now subject to reconsideration.

In Aldana's scale-free model [11], where the output degree distribution follows a power-law 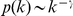, where 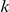 is the variable node degree, this phase transition is obtained by setting 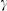 around 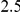. In our case, we use real-life networks and not hand-made ones, and thus, we cannot tune any property of the network topologies to obtain the desired critical regime, or even to identify the regime of one of our network. Instead we use a dynamical property of the whole system which is represented by Derrida plots, proposed by Derrida *et al.* [8], used by Kauffman [22], and widely accepted as a method of discriminating the regime in which RBN-like dynamical systems evolve.

**Figure 2:**
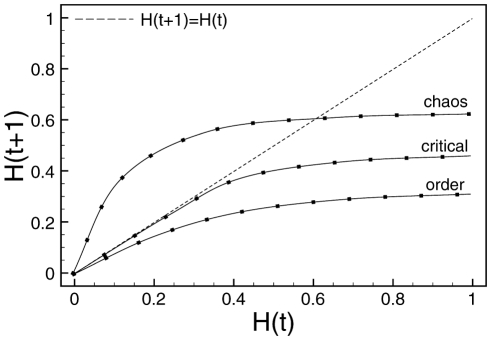
Derrida plot. Derrida plot of the original RBN model (see text).

This representation is meant to illustrate a convergence versus a divergence in state space that can in turn help characterizing the different regimes. It uses the Hamming distance 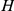, defined as the normalized number of positions that differ when comparing two (binary) strings. These plots show the average Hamming distance 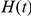 between any two states 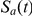 and 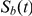 and the Hamming distance 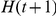 of their respective subsequent states 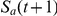 and 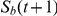. Figure 2 shows a typical instance of Derrida plots and curves for all three regimes. Derrida plots of systems in the chaotic regime will remain above the main diagonal 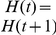, i.e. their distance tends to increase during a certain time, then cross the main diagonal from above.

A Derrida plot is the graphical representation of the mapping that relates the size of the perturbation avalanche on a RBN model at two consecutive time steps. In a mean-field approximation, this mapping can be shown to be a smooth continuous monotonously increasing function, with only one stable fixed point that determines the dynamical regime in which the RBN operates. The value of this fixed point depends only on the slope (derivative) at the origin of the mapping, often called the *average network sensitivity* [23].

However, this is an approximation, as the mean-field originally applies to the thermodynamic limit with 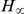, which is not reachable, not even in principle, in real biological networks, as these are intrinsically made of too few genes. Even the notion of derivative of the Derrida plot is ill-defined since the Hamming distances are discrete values. Therefore analytical results relating the slope at the origin of the Derrida plots to the regime, which hold in the mean-field approximation in the thermodynamic limit, cannot be relied upon in small biological systems. Nevertheless, the Derrida plots we will derive empirically later in this work show a behavior that is qualitatively similar to the one found in traditional RBNs. Therefore, we use them in this section as first approximation to distinguish ordered, critical, and chaotic regimes. Then, in order to account for the particular case of real-life biological networks, we propose to use a new metric: *c*riticality distance as defined above. The criticality distance takes into account the fate of perturbations of various strengths and can be used for finite networks in which the notion of derivative is not well defined.

Systems in the critical regime remain *on* the main diagonal at the beginning and then stay below the main diagonal. Ordered systems remain under the main diagonal at all times. In other words, systems in the critical regime we are interested in, which lies in the ordered regime at the edge of chaos, are characterized by Derrida curves that remain as close as possible to the main diagonal before diverging.

As said before, the modules presented in this work show no sign of a long-tailed degree distribution. Therefore, we are very far from the limit in which there is a true phase transition with 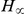. In this framework, we use the Derrida mapping as a first approximation to investigate the dynamical behavior of the two models under random update functions (RUFs). As we can not be certain that the mean-field assumptions hold, we portray the state space of the two systems.

In these two RBN models it is obviously not possible to tune the connectivity parameter 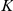, since the interconnection topology is fixed by experimental data. However, when the systems' dynamics are driven by the original nodes' RUFs, the probability 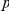 could still allow the two models to be in different regimes. The number of all possible states for a given RBNs, i.e. with a single set of RUFs, is 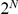, where 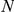 is the number of genes in the system. In our case, 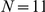, therefore there are 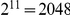 possible states. The set of possible RUFs, even for a reasonably small subset of genes like the present, makes exhaustive enumeration impossible for original RBNs. Therefore, we resorted to statistical sampling by performing numerical simulation of 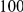 different sets of RUFs for each value of 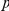. At first, 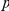 varies in the interval 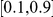 by steps of 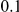. Having identified the values of interest 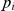, we narrowed the interval to 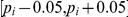 with a finer step of 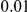 to identify the values 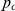 that are closest to the critical region.

**Figure 3:**
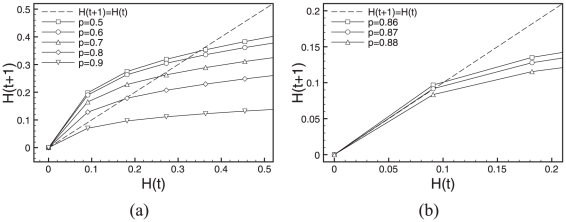
Derrida plots of RUFs for ES cell. (a) 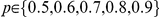 (curves for 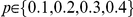 are not reported as RUF rules are symmetrical), and (b) only values close to the critical gene expression value 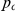 are investigated with refinement steps of 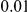. Please note the two different scales in the axes.

**Figure 4:**
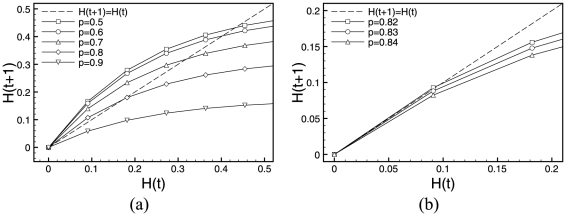
Derrida plots of RUFs for yeast cell. (a) 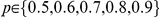 (curves for 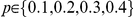 are not reported as RUF rules are symmetrical), and (b) only values close to the critical gene expression value 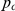 are investigated with refinement steps of 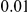. Please note the two different axes scales in the figures.

Figures 3 and 4 show Derrida plots with steps of 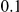 (a) and the finer version (b), where we adapted the scale to best show the regions of interest with a step of 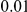. As there are only 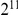 possible states, we computed average Hamming distances over exhaustive enumeration of all possible states. In other words, we identified all pairs of states 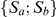 that are at a distance 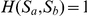 and computed the average Hamming distance of their subsequent states 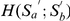, and then moved on to a distance 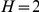, 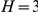,…, 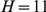.

For the two regulatory network models, Figures 3 and 4, depict the Derrida curves according to their values of 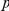, as the RUF functions are symmetrical for values of 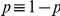. If not for sampling reasons, pairs of curves would superimpose, and therefore, to facilitate the interpretation of the results, we only plot curves for values of 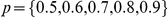. As shown in the Figure 3a for transcriptional regulatory network in ES cell, the interesting values of 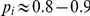, and symmetrically, 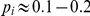. These are the values we chose to investigate with finer steps in Figure 3b, revealing that in the case of ES cells the critical threshold value is close to 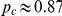, and symmetrically 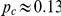.

Also for the case of the yeast cell-cycle regulatory network in Figure 4a, we identified 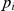 to approximately the same values, more finely investigated in Figure 4b, where again 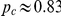, symmetrically 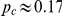.

#### Criticality Distance

**Figure 5:**
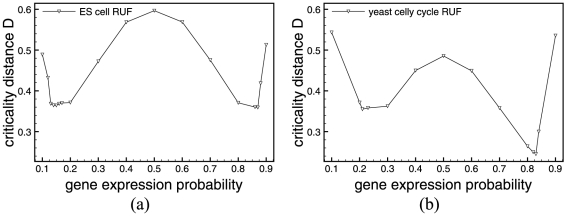
Minimal Criticality Distances of Random Update functions. Criticality distances computed for each gene expression probability of RUF for both ES cells (a) and Yeast (b) from the Derrida plot/HD data.

If we consider synthetic systems, such as the ones producing Figure 2, where the system's properties define the regime, the slope of the curve at the origin is an adequate measurement to confirm the system's dynamical regime. Nevertheless, if we take the example of real-life systems we conclude that this metric alone is not sufficient to capture the divergence of the system over the entire range of Hamming distances. Indeed, two curves with identical slopes at the origin, thus in the same regime, can then diverge from each other. In natural systems, we cannot fine tune the properties in order to set the regime to critical, we merely try to identify in which cases we are closest to the critical regime. If we take the example the Derrida curves of two systems in the ordered regime diverging despite identical slopes at the origin, we still can assume that the system with the curve closest to the main diagonal is the one closer to the critical regime. In order to address this and try and get a feel for the systems behavior over the whole spectrum of 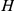, we propose a single numerical value that characterizes the distance of a system's Derrida plot to the main diagonal. This new *normalized criticality distance* (D) takes into account that the closeness to the main diagonal is more important for smaller values of 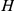. The normalized criticality distance is obtained as follows:
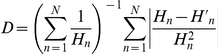
where 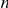 varies over all possible values of the Hamming distance 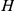, therefore 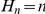. 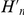 is the average Hamming distance of the subsequent states of all couple of states at distance 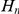. The closer 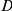 is to zero, the closer our system is to the critical regime, and therefore, the more interesting it is for in the context of this work. We use this new metric, in addition to the Derrida plot, to determine for which parameter sets the investigated systems are in the *critical* regime. We suggest to use both metrics together, as Derrida plots discriminate whether a system is in the ordered or chaotic regime, while the criticality distance quantifies how close the previously determined regime is to the critical phase transition. Figure 5 below shows how the minimal value 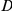 evolves with respect to the probability of gene expression 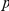 of each nodes' internal update function.

In Figure 5, we show 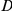 values for gene expression probabilities 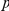 by steps of 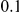 over the entire spectrum. We refine the study around the minima with steps of 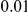. Although the random update functions of RBNs are symmetrical with respect with 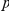 and 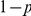, the solution space of all possible Boolean functions is much too big to be exhaustively explored, we therefore chose to sample 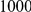 different random update functions and average out the results. The fluctuations observed in Figure 5b can be explained by the sampling of this particular simulation.

For both networks the results obtained for 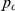 using the Derrida plots, although not equal, agree with that found using the criticality distance. In the case of ES cells, the approximate minimal 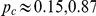 and for yeast cell cycle, 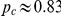. Again, the fluctuation in the function's symmetry are due to a sampling effect.

### Modeling the Yeast and the Embryonic Stem Cells Regulatory Networks

In the original RBN model, each node was assigned a deterministic distinct random update function (RUF). Even if their exact values are unknown, it is clear that gene update functions should not be random. Recent results suggest that genes expression rests on the combined effect of regulatory inputs that can have either an *activating* (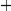) or *repressing* (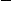) action on their target genes. Nowadays, it is believed that the subset of Boolean functions approximating the genes' regulation can be of two types, depending on the genes and the system at hand: canalizing combinatorial function [24] or additive function [25]. In this work, we focus on the latter to better match certain real-life regulation mechanisms, where the influence of the genes upstream of the target, along with its own current activity state, could be summed in a way that takes into account the *activating* or *repressing* effect of each influencing node. Evidence of canalizing functions (e.g. *XOR*) can be found in modules of GRNs and have been studied throughly [23], [26]. In this work we analyze the effect of a different type of Boolean update function that was proven to regulate a majority of genes in specific cases of GRNs. Indeed, many studies (see e.g. [27]–[29]) have shown that models in which the contribution of different transcription factors to the regulation of a target gene is treated additively can successfully explain a significant part of the variation in gene expression. Recently an additive model was shown [30] to explain up to 65% of the gene expression variation in the same biological context (embryonic stem cells) as one of our networks.

Li *et al.* [13] proposed a simple additive dynamical rule that characterizes the temporal evolution of the state variable. They consider that both the activating and repressing factors have the same weight, and thus, the state of a target gene at the next time-step 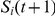 will be: *active* (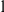) if it receives a majority of *activating* components from already active genes, *inactive* (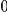) it receives a majority of *repressing* components, or the state of the target gene will remain unchanged in the case the number of *activating* and *repressing* inputs are equal. Inspired by their work, we propose an update function shared by all genes that takes into account the fact that *activating* and *repressing* components could have uneven effects. In this case, a gene could require a majority by more than one active input to switch states. Therefore we introduce a *threshold* value 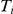, for the 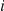-th gene, which has to be reached in order for a gene to become active. The principal reason that motivates this further investigation on the model proposed by Li *et al.* is the presence in most of recent models of a kind of threshold value for the activation of genes (see, for example the review [25]). A gene's activation state at the next time-step 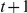 is now given by:
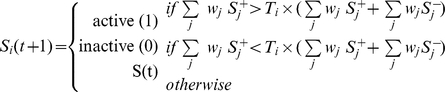
Where 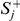 (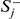) is the state of an activator (repressor) of the target gene, and 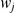 is the weight of each specific edge (i.e. regulating effect). In this first study, and in the absence of actual system's specific quantified values of either 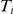 or 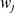 we assume a common, yet variable, threshold value 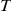 for all genes, and a identical weight 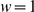 for all connections. Moreover, as we are studying modules (sub-networks) of GRNs, and not complete ones, some genes of our model might not have any repressors. Thus, if activated, these genes remain expressed permanently. In the case where an active gene has no repressor at all, we automatically repress it at the next time-step, simulating a gene-product decay rate that exceeds the production time. The update function presented in this section is equivalent to Li's in the case where 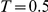. We call our model for update function the Activator Driven Additive function (ADA).

It can be easily proven that all rules in this class correspond to a subset of the RUFs [31]. In fact, once given, for each node, the *activating* and *repressing* effects of its neighbors, for each possible configuration of the neighborhood, the lookup table of the corresponding additive rule of the node can be constructed. In this form it can be easily recognized as an instance of the RUFs in the original Kauffman's RBN model. Therefore, by using ADA functions with different 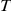-parameter values in a RBN model, we are exploring the behaviors of a subset of classical RBNs.

Another interesting implication of this update function is that under this assumption the synchronous timing of the events coincides with the semi-synchronous topology driven update scheme we recently investigated in [10]. This update sequence is neither fully synchronous nor asynchronous, but rather takes into account the sequence in which genes affect each other. In this scheme, only the activation of an activator or a repressor will have an effect on the list of nodes to be updated at the next time-step. Thus, the set of all nodes that have to be updated in each time step is formed by those genes that have at least an in-neighboring active gene, even when a RUF is used to evolve the model. On the other side, when an ADA function is employed, in a synchronous timing of the update events, a node is actually updated only if it has at least an in-neighboring active gene.

## Results

### Regimes Characterization in Real-Life Networks

Just as the probability 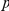 can change Kauffman's original systems' regime from chaotic to ordered for a given connectivity 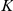 and set of RUFs, the 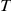-parameter in our ADA model can change its regime. In the following section, we show for which values of 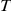 our model of real-life topology based Boolean networks using ADA exhibit a phase transition, and compare the dynamics of the two update functions.

As discussed previously, the space of all possible states for a given RBNs is 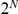, where 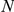 is the number of genes in the system. In our case, 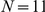, therefore there are 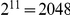 possible states. In the case of ADA, where all nodes share the same Boolean update function, exhaustive enumeration is possible. At first, we let the threshold 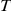 parameter vary in the interval 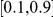 by steps of 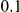. After identifying the values of interest 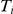, we narrowed the interval to 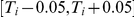 with a finer step of 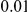 to identify as precisely as possible the values 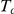 that are closest to the critical region. As there are only 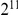 possible states, and thus the maximum Hamming distance 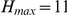, we computed average Hamming distances over exhaustive enumeration of all states. In other words, we identified all pairs of states 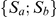 that are at a distance 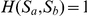 and computed the average Hamming distance of their subsequent states 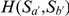, and then moved on to a distance 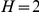, 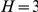, , 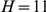.

**Figure 6:**
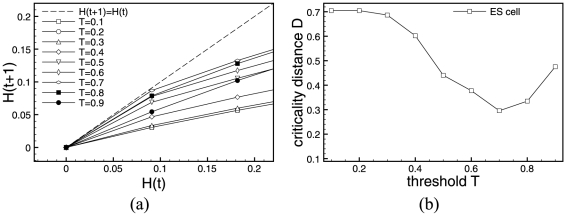
Critical threshold. Derrida plots and Criticality Distances of Activator Driven Additive functions for the mouse embryonic stem cell regulatory network.

**Figure 7:**
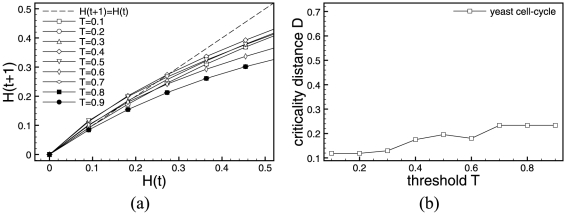
Critical threshold. Derrida plots and Criticality Distances of Activator Driven Additive functions for the yeast cell-cycle regulatory network.

The left-hand sides of Figures 6 and 7 show Derrida plots with steps of 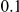. The regions of interest for the 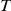 values are 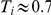 for ES cells. Let us note that in the ADA case, contrary to RUF, update rules are not symmetrical with respect to 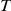 and 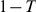. For yeast cell-cycle, we see two regions worth investigating 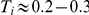 and 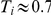. The in-depth examination of ADA simulation results for values of the 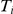 parameter demonstrate that results become undistinguishable (thus, figures are not shown) when the step between 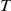 values becomes small. This is due to the fact that the ADA function is less sensitive to 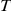 for genes with a low input degree. In the case of ES cells, 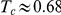. In the case of yeast, the Derrida plot suggest two values of 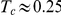 or 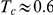. In this last value of 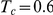, curves for several very close values of 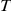 coincide. Now, we use the criticality distance to chose the 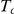 closest to the phase transition: 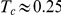. We report the Derrida plots and criticality distances for both systems in Figures 6 and 7.

In order to increase the readability of the results, we chose not to include the results of the refined simulations with steps of 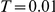. Nevertheless, summarize these results in the Table 2.

**Table 2:** Real-life networks critical values.

	RUF 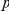 values			ADA 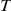 values		
	order	critical	chaos	order	critical	chaos
ES cell	0.1, 0.9	0.13, 0.87	0.5	0.2	0.68	N/A
yeast cell	0.1, 0.9	0.17, 0.83	0.5	0.9	0.25, 0.6	0.4

For systems under RUF, we show the function's gene expression probability 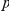 values for all three regimes, for both ES cells and Yeast cell-cycle. In the case of ADA, we give threshold values 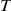 also for all three regimes and both studied networks.

From these results, we observe that the ADA-thresholds 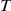 have comparable values in the two GRNs studied in this paper: 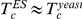. The same applies to the probabilities 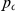 of gene expression in RUF.

#### Validation of the Model on a Network with Known Update Rules

The two partial GRNs previously presented are practical RBN models of dynamical regulatory interaction networks as they are small enough to study exhaustively all 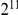 possible states of the system. Therefore, we can fully define the update functions for each node and every possible input combination. Yet, in these particular cases, we have nothing to compare these functions against. In order to validate ADA update functions, we used another regulatory network presented in [15].

In this work, Li *et al.* define a dynamic Boolean model of plant guard cell abscisic acid (ABA) signaling. This hormone allows plants to adjust water conservation within the organism. In the original model presented, the regulatory network is made of 42 cellular components. For each of these components, in addition to their connections, the authors defined the Boolean function that decides the state of each component at the next time-step. This new information can help us assess the validity of the ADA update function.

#### ABA Network Reduction

Another helpful feature of the ABA regulation network is that 4 of the components have a predefined Boolean value and those do not have an update function attached to them. This allows us to replace these *constant* components (i.e. that are assigned a Boolean value) in the update function of the 38 remaining ones, and then to replace some more that become constant. For example:
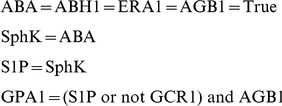
becomes after simplification:
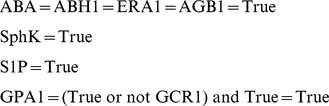
Following this logic, the fully simplified ABA network becomes:
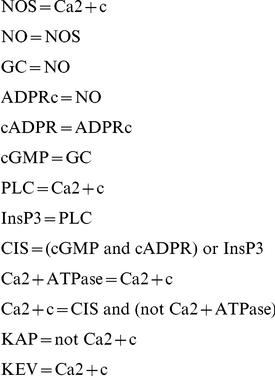



This new simplified network is reduced to only 13 components and it is therefore possible to enumerate all possible 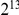 states.

#### Determining the ADA model's regime

**Figure 8:**
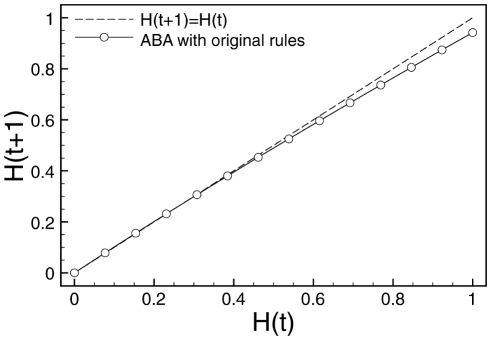
Regime of the ABA model. Derrida plot of the simplified ABA model with the original real-life update functions.

In order to determine the regime in which the ABA model evolves, we used the Derrida plots, shown in Figure 8. The criticality distance is not as useful in this instance, as there is no comparison to be made.

**Figure 9:**
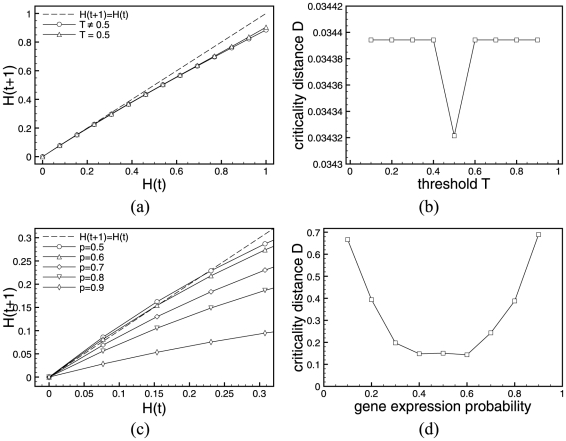
Critical threshold and gene expression probability. Derrida plots (a)(c) and criticality distance vs. the threshold (b), respectively gene expression probability (d). The upper row (a)(b) shows the ABA system where the original rules have been replaced by ADA update function. In the lower row (c)(d), rules have been replaced by RUF and results are averaged over 100 random rules sets.

The Derrida plot of the ABA model with real-life update functions depicted in Figure 8 clearly shows that the system evolves near the critical regime. Therefore, we use the Derrida plots and criticality distance 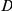 to determine the critical values 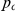 of RUF, respectively 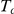 for ADA, when each of these function families is substituted in the simplified ABA model. In the case of RUF, we average out the results over 100 sets of different update functions. Derrida plots for ABA system with ADA update over the full scope of 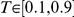 values together with the corresponding evolution of the criticality distance are shown in Figures 9(a) and 9(b). The plot for RUFs over the same range of 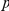 values is depicted in Figures 9(c) and 9(d).

From the analysis of the figures above, we observe that in the case of ADA (Figure 8(b)), there are only two values of the criticality distance, one for 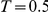 and a larger one for 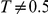. The first one is the closest to the original ABA model critical regime when 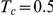. Arguably, there is very little difference in the system's dynamical behavior as 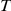 changes. This is due to the fact that 11 out of the 13 genes in the ABA model have a single input upstream gene. The system is close to critical for any value of 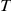 (see Derrida plot in Figure 9(a)) and therefore rather insensitive to the parameter 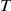.

In the case of RUF (Figures 9(c) and 9(d)), the closest gene expression value to the regime of interest is 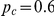.

#### Comparing ADA, RUF and real-life functions

Using the ABA network described above, we have fully defined each node's lookup table according to its real-life function. Subsequently, we have replaced the original update functions of each node with ADA functions and 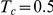 to define the new lookup tables. This allows us to compare in a very straight forward manner how close ADA is to this specific case real-life activations. In addition, we have also replaced the set of node functions by a sample of 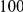 RUFs and 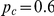, and averaged out the results. In order to keep the measurements simple, we have computed the normalized Hamming distance between the real-life ABA function and ADA, or respectively RUF. Each node's lookup table size is 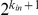, where 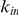 is the node's incoming degree. Therefore, the added size of all nodes lookup tables is 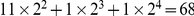.

In the case comparing ABA and ADA, the Hamming distance 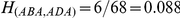, which means that the functions outcome overlap by more than 90%. Therefore, we can assume that in the specific system described above, the real update function is very close to an additive one. We contrast this result with that comparing RUF to ADA, where the average Hamming distance over the 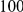 RUF sets is 
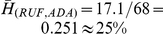
. These results show that in this particular case, ADA is significantly closer to the real-life ABA function than a random function. Although this finding cannot be generalized at this time, it suggests that at least in some cases, the ADA function ought to be closer to the real-life update function of a regulatory network system.

### Dynamical Behaviors of Real-Life Regulatory Networks

A key notion underlying the behavior of deterministic discrete dynamical networks is that they organize their state space into a set of basins of attraction. When a discrete dynamical network perspective is used to investigate genetic regulatory networks behaviors, understanding how the range of stable cell types can exist with identical genes becomes clearer. Different attractors into which network dynamics settles from various initial states can be seen as cell types or modes of growth for unicellular organisms, while the trajectories leading to attractors can be seen as the pathways of differentiation.

To better understand real-life regulatory networks, it is not enough to qualify their regime by Derrida mapping only. It is therefore useful to portray their state space. Several measures have been proposed to characterize the state space of a dynamical network by Wuensche [32]. Of particular interest are the number and lengths of the attractors in the state space, together with the sizes of the basins of attraction. Finally, according to Wuensche [32]: “high leaf density, high branching, short transients, and small attractor cycles indicate order”. Leafs are states of the state space that do not have any predecessor, while transient times and branch lengths are the time steps (i.e. number of states) necessary from a state and a leaf respectively to reach its attractor. If it is generally accepted that entire GRNs operate in the critical regime, it is also clear that modules, or sub-networks, function in different dynamical regimes. It is therefore useful to study the dynamical behavior of our systems in all three regimes.

In the following sections, we study the dynamical behavior of ES cells and yeast cell-cycle separately. In both cases we compare results obtained using the ADA update function and those obtained using random update functions found in classical RBN. In the case of ADA, where the update function is unique, we exhaustively enumerate the entire state space a single time. On the contrary, in the case of RUF, we sample 1000 different sets of rules unique to each gene. This is the reason why there is standard error information only in the RUF case.

#### Simulation of the Embryonic Stem Cell Regulatory Networks

Using the model of mouse embryonic stem cell regulatory networks with ADA, we constructed systems in the two available regimes: ordered and critical. Indeed there is no value of 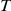 that clearly puts the system in the chaotic phase. Therefore, we use 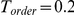 and 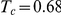 for the unique ADA model. On the other hand, we built 1000 RUF models of the ES systems, with as many different sets of unique rule in each gene. In the RUF case, 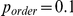, 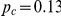, and 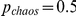.

**Figure 10:**
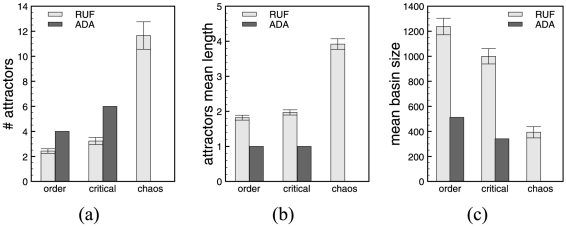
Attractor space analysis. Numerical simulation results for the ES model. (a) Attractors (average) number, (b) attractors average lengths, and (c) the average basin of attraction size. The statistics are computed on samples of RUF systems, hence the standard error bars, and exhaustively on ADA systems.

Figure 10 shows the numerical simulations results in terms of number of attractors for ADA, respectively average number of attractors for RUF, average attractor lengths, and average basin size. Error bars represent the standard error.

In the case of ES cells, we observe an increase in the number and length of the attractors, explaining the shrinkage in the basins' sizes, as the systems are getting more chaotic. This agrees with previously obtained results on lager network models [10], and it is aligned to Kauffman's conjecture that the biggest increase in this characteristic should happen in the chaotic regime. In the ADA case, we find more attractors of shorter length than in the case of RUF, almost all being point attractors. Between RUF and ADA models, mean transient times and mean branch lengths do not differ, showing a tendency to considerably increase in the chaotic regime. In ordered and critical regimes, the mean branch lengths and transient times are very short (smaller than 2 states), explaining the close to 1 probability of having leaf-states. These measures suggest that considering ADA functions we are focusing on a more biologically interesting and plausible subset of RUFs.

#### Simulation of the Yeast Cell-Cycle Regulatory Networks

**Figure 11:**
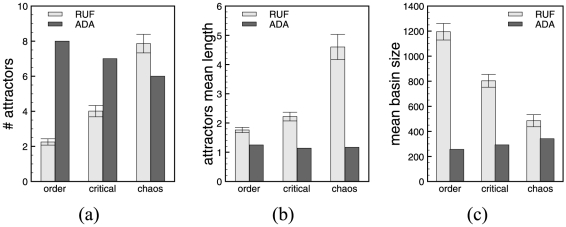
Attractor space analysis. Numerical simulation results for the yeast model. (a) Attractors (average) number, (b) attractors average lengths, and (c) the average basin of attraction size. The statistics are computed on samples of RUF systems, hence the standard error bars, and exhaustively on ADA systems.

In contrast with the mouse embryonic stem cell regulatory network, the yeast cell-cycle one with ADA can be found in all three regimes. Simulations for networks in the different regimes and with both RUF and ADA functions have been performed in the same manner as for the ES model. Figure 11 shows the numerical simulations results in terms of number of attractors for ADA, respectively average number of attractors for RUF, average attractor lengths, and average basin size. Error bars represent the standard error.

In this case, while RUF behaves as expected with a growth in the number of attractors as the systems moves to chaos, surprisingly, the opposite behavior can be observed when ADA functions are employed. When considering basin entropy 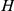, as expected RUF models tend to show higher values in critical and chaotic regimes. On the contrary, ADA systems' entropies dramatically drops from 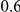 in the ordered regimes to 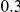 in the critical one and to 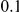 when chaotic. This behavior could be expected if the decrease in the number of attractors was significant, which is not the case here. Therefore, we are witnessing the dominance in their basin sizes of a small number of attractors. These yeast cell-cycle systems thus show a biologically interesting feature, compatible with the assumption that attractors correspond to cell-cycles. Mean attractors lengths, basin sizes, transient and branch lengths, and probability of leaf-states show an identical behavior to that of ES cell regulatory networks.

### Resilience to Small Perturbations

Failures in systems can occur in various ways, and the probability of some kind of error increases dramatically with the complexity of the systems. They can range from a one-time wrong output to a complete breakdown and can be system-related or due to external factors. Living organisms are robust to a great variety of genetic changes, and since RBNs are simple models of the dynamics of biological interactions, it is interesting and legitimate to ask questions about their fault tolerance aspects.

Kauffman [33] defines one type of perturbation to RBNs as “gene damage”, that is the transient reversal of a single gene in the network. These temporary changes in the expression of a gene are extremely common in the normal development of an organism. The effect of a single stimulus can transiently modify the activity of a gene, resulting in a growing cascade of alternations in the expression of genes influencing each other. Although not agreed by all, some believe this to be at the origin of the cell differentiation process and guides the development.

The precise structure of attractor basins is of interest as it may reflect the stability of cell types to perturbation. A set of similar states can be specified for example that differ by one bit from a reference state (a Hamming distance of one). The distribution of the set across the basin of attraction held indicates the network's response to a one bit perturbation to its current state of activation. The dynamics of the system might remain in the same basin or flip to a different basin.

For both the mouse embryonic stem cell and the yeast cell-cycle regulatory networks, we computed 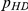, the probability that two states at Hamming distance of one belong to the same basin of attraction. The two network models are studied both with RUF and ADA update functions. In the case of ES cells, both update functions show identical 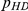 in the ordered regime, while in the critical region 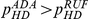. This same relationship holds in the critical regime of the yeast cell-cycle system, even though 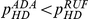 in the order. Therefore, in the critical region the ADA function shows higher probability, thus better resilience to single-gene perturbations. These numerical results are shown in the Table 3.

**Table 3:** Resilience to small perturbations.

	RUF	ADA
ES cell	0.83	0.9
yeast cell cycle	0.76	0.86

Probability 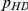 that two states at Hamming distance of one belong to the same basin of attraction for both systems under RUF and ADA. The resilience to faults of ADA is consistently superior.

## Discussion

Taking into account recent years' advances in the field of cellular biology, we have proposed to identify under what conditions Kauffman's hypothesis that living organism cells operate in a region bordering order and chaos holds. This property confers to organisms both the stability to resist transcriptional errors and external disruptions, and, at the same time, the flexibility necessary to evolution. We studied two particular cases of genetic regulatory networks found in literature in terms of complex dynamical systems derived from the original RBN model. Therefore, we compared the behavior of these systems under the original update function and a novel additive function that we believe is closer to the actual role of living organisms.

The proposed functions, here called Activator Driven Additive (ADA), correspond to a subset of all possible Boolean functions of the original random Boolean network model. Moreover, using this set of update rules, the synchronous timing of the events coincides with the semi-synchronous topology driven update scheme we recently investigated. This update sequence is neither fully synchronous nor asynchronous, but rather takes into account the order in which genes affect each other. This new update function, although very basic, shows excellent results in the case of biological organisms' GRN models, and have the advantage that the results are reproducible, whereas this is not true with random update functions that are, by definition, different with every simulation.

In order to investigate the dynamical behaviors of this new model, we visualized the phase transition between order and chaos into the critical regime using Derrida plots. We also proposed a new measure, the criticality distance, that allows to numerically discriminate between different regimes by the method implemented by Derrida plots. The two measures are complementary and should be used in conjunction. In fact, the criticality distance quantifies how close the system's regime determined by Derrida plots is to the critical phase transition.

Simulation results on two real-life genetic regulatory networks, the yeast cell-cycle and the mouse embryonic stem cell, show that there exist parameter settings in both update functions that allow the systems to operate in the critical region, and that these values are comparable in the two case studies. Both Derrida plots and criticality distances agree on the numerical values of the parameter for which the transition into the critical regime takes place. To better understand real-life regulatory networks, it is not enough to qualify their regime. The state spaces of the two real-life GRNs is portrayed using RBN-specific statistical measurements, confirming that the two systems operate at the edge of chaos. Moreover, in the critical regime, we show that ADA systems exhibit superior tolerance to transient perturbations than classical RBNs.

To validate ADA update functions, we used another bio-chemical regulation network operating near the critical regime (as confirmed by Derrida plot). For each node of this network, in addition to their connections, the authors defined the Boolean function that decides the state of each component at the next time-step. This new information can help us to assess the validity of the ADA update function. These results show that in this particular case, ADA is significantly closer to the real-life function than a random function. This also comforts us that, at least in some cases, the ADA function ought to be closer to the real-life update function of a regulatory network system.

A crucial step in order to bring the model closer to biological soundness could consist in considering different threshold values for each node and different weights for each regulatory edge. The resulting nodes' ADA update functions could drive the model toward more realistic patterns of gene regulation dynamics. Moreover, we consider combining the canalizing combinatorial functions and the additive functions within the same model. Finally, this new model should be validated on larger gene regulatory networks of different biological organisms.

## References

[1] Kauffman SA (1969). Metabolic stability and epigenesis in randomly constructed genetic nets.. J Theor Biol.

[2] Langton CG (1990). Computation at the edge of chaos: Phase transitions and emergent computation.. Physica D.

[3] Bak P, Tang C, Wiesenfeld K (1988). Self-organized criticality.. Physical Review A.

[4] Kauffman SA (1993). The Origins of Order.

[5] Levine M, Tjian R (2003). Transcription regulation and animal diversity.. Nature.

[6] Kitano H (2004/11//print). Biological robustness.. Nat Rev Genet.

[7] Sethna JP, Dahmen KA, Myers CR (2001/03/08/print). Crackling noise.. Nature.

[8] Derrida B, Pomeau Y (1986). Random networks of automata: a simple annealed approximation.. Europhysics Letters.

[9] Darabos C, Giacobini M, Tomassini M (2007). Semi-synchronous activation in scale-free boolean networks.. Advances in Artificial Life.

[10] Darabos C, Tomassini M, Giacobini M (2009). Dynamics of unperturbed and noisy generalized boolean networks.. Journal of Theoretical Biology.

[11] Aldana M (2003). Boolean dynamics of networks with scale-free topology.. Physica D.

[12] Balleza E, Alvarez-Buylla ER, Chaos A, Kauffman S, Shmulevich I (2008). Critical dynamics in genetic regulatory networks: Examples from four kingdoms.. PLoS ONE.

[13] Li F, Long T, Lu Y, Ouyang Q, Tang C (2004). The yeast cell-cycle network is robustly designed.. Proceedings of the National Academy of Sciences of the United States of America.

[14] Darabos C, Giacobini M, Tomassini M, Provero P, Cunto FD, et al GK (2009). Are cells really operating at the edge of chaos? a case study of two real-life regulatory networks.. Advances in Artificial Life, ECAL2009.

[15] Li S, Assmann SM, Albert R (2006). Predicting essential components of signal transduction networks: A dynamic model of guard cell abscisic acid signaling.. PLoS Biol.

[16] Chen X, Xu H, Yuan P, Fang F, Huss M (2008). Integration of external signaling pathways with the core transcriptional network in embryonic stem cells.. Cell.

[17] Mansergh F, Daly C, Hurley A, Wride M, Hunter S (2009). Gene expression profiles during early differentiation of mouse embryonic stem cells.. BMC Developmental Biology.

[18] Stoll G, Rougemont J, Naef F (2007). Representing perturbed dynamics in biological network models.. Phys Rev Lett E.

[19] Bower JM, Bolouri H (2001). Computational Modeling of Genetic and Biochemical Networks.

[20] Smolen P, Baxter D, Byrne J (2000). Modeling transcriptional control in gene networks–methods, recent results, and future directions.. Bulletin of Mathematical Biology.

[21] Hasty J, McMillen D, Isaacs F, Collins JJ (2001/04//print). Computational studies of gene regulatory networks: in numero molecular biology.. Nat Rev Genet.

[22] Kauffman S (2003). Understanding genetic regulatory networks.. International Journal of Astrobiology.

[23] Shmulevich I, Kauffman SA (2004). Activities and sensitivities in boolean network models.. Phys Rev Lett.

[24] Perissi V, Jepsen K, Glass CK, Rosenfeld MG (2010). Deconstructing repression: evolving models of co-repressor action.. Nat Rev Genet.

[25] Alon U (2007). Network motifs: theory and experimental approaches.. Nat Rev Genet.

[26] Kauffman S, Peterson C, Samuelsson B, Troein C (2003). Random boolean network models and the yeast transcriptional network.. Proceedings of the National Academy of Sciences.

[27] Keles S, van der Laan M, Eisen MB (2002). Identification of regulatory elements using a feature selection method.. Bioinformatics.

[28] Conlon EM, Liu XS, Lieb JD, Liu JS (2003). Integrating regulatory motif discovery and genome-wide expression analysis.. Proceedings of the National Academy of Sciences of the United States of America.

[29] Bussemaker HJ, Li H, Siggia ED (2001). Regulatory element detection using correlation with expression.. Nature genetics.

[30] Ouyang Z, Zhou Q, Wong WHH (2009). Chip-seq of transcription factors predicts absolute and differential gene expression in embryonic stem cells.. Proceedings of the National Academy of Sciences of the United States of America.

[31] Hassoun MH (1995). Fundamentals of artificial neural networks.

[32] Wuensche A, et al PA (1989). Genomic regulation modeled as a network with basins of attraction.. Pacific Symposium on Biocomputing.

[33] Kauffman SA (2000). Investigations.

